# The Detection and Differentiation of Pigeon Adenovirus Types 1 and 2 via a High-Resolution Melting Curve Platform

**DOI:** 10.3390/microorganisms13061331

**Published:** 2025-06-07

**Authors:** Shuyu Chen, Wenyu Zhang, Zhiwang Tang, Tingting Lu, Chunhe Wan, Wensong Jin, Jiayu Li

**Affiliations:** 1 School of Life Sciences, Fujian Agriculture and Forestry University, Fuzhou 350002, China; chenshuyu0726@163.com (S.C.); 13340922216@163.com (W.Z.); tangzw0820@163.com (Z.T.); 19855953983@163.com (T.L.); jinws@fafu.edu.cn (W.J.); 2 Institute of Animal Husbandry and Veterinary Medicine, Fujian Academy of Agricultural Sciences, Fuzhou 350013, China; 3 Fujian Key Laboratory for Avian Diseases Control and Prevention, Fujian Academy of Agricultural Sciences, Fuzhou 350013, China

**Keywords:** PiAdV-1, PiAdV-2, qPCR-HRM platform, detection and differentiation, epidemiological investigation, coinfection

## Abstract

Two main adenoviral diseases have been described in pigeons: pigeon adenovirus type 1 (PiAdV-1) and pigeon adenovirus type 2 (PiAdV-2), which belong to the genus *Aviadenovirus* under the family *Adenoviridae*. PiAdV-1 and PiAdV-2 are highly pathogenic to pigeons, leading to considerable losses worldwide. To date, there is little information on the epidemiological distribution of PiAdV-1 and PiAdV-2 in pigeons due to the lack of detection and differentiation platforms for these two viruses. High-resolution melting technology (HRM) has been widely used for developing detection and differentiation platforms, with the melting profile based on the GC content in the real-time PCR (qPCR-HRM) system. This study designed and synthesized a pair of specific primers on the basis of the characteristic variations of the 52K genes of PiAdV-1 and PiAdV-2, then the detection and differentiation qPCR-HRM platform was established after conditional optimization. The results showed that this method had good specificity; it could only specifically detect PiAdV-1 and PiAdV-2, with no cross-reaction with other pigeon-origin pathogens that occur in pigeons. This method had high sensitivity, with the lowest detection limits at 57 copies/µL (for PiAdV-1) and 56 copies/µL (for PiAdV-2). This method had good intra-group and inter-group coefficients of variation, both of which were less than 1.5%. Field samples for the epidemiological surveillance and investigation data of PiAdV-1 and PiAdV-2 were checked. We found only PiAdV-2-positive samples in meat pigeons, but the percentages of PiAdV-1-positive, PiAdV-2-positive, and coinfection-positive samples among the racing pigeons were 5.71%, 14.29%, and 2.86%, respectively. To our knowledge, this is the first report for the simultaneous detection and differentiation of PiAdV-1 and PiAdV-2 using the qPCR-HRM platform. Our study also provided evidence of PiAdV-1 and PiAdV-2 coinfection in racing pigeons, but further studies are needed.

## 1. Introduction

In recent years, the racing pigeon and meat pigeon breeding industry has developed rapidly and has become the fourth largest poultry industry after chickens, ducks, and geese in China [1,2]. Pigeon adenovirus (PiAdV) infection has been occurring in young pigeons and is associated with high losses. Owing to various factors, such as the training of racing pigeons and cross-regional trade, pigeon adenoviruses have been widely prevalent worldwide [3,4,5,6]. Two main adenoviral diseases have been described in pigeons, pigeon adenovirus type 1 (PiAdV-1) and pigeon adenovirus type 2 (PiAdV-2), on the basis of their different clinical characteristics [7,8]. PiAdV-1 affects mainly young birds and the main symptoms of sick pigeons include diarrhea, vomiting, and weight loss. The clinical symptoms are significantly similar to those of young pigeon disease syndrome (YPDS). Histologically, microscopic examination revealed the evidence of inclusion bodies from intestinal epithelial cells and liver cells [9,10,11,12,13]. PiAdV-2 can affect pigeons of all ages, and the typical characteristics of the disease are sudden death and extensive liver necrosis in the sick birds. The clinical symptoms are not obvious, and occasionally, pigeons may present with vomiting and yellow watery discharge [14,15]. Usually, pigeons that are typically infected with only PiAdV have a low mortality rate. However, pigeons that are coinfected with Escherichia coli [16], circovirus [17], herpesvirus [18], influenza viruses [19], and so on [20,21,22], can significantly increase the mortality rate, which makes early identification and diagnosis of the disease difficult.

Pigeon adenovirus is a non-enveloped linear dsDNA virus that belongs to the genus *Aviadenovirus* in the *Adenoviridae* family. The members of the genus *Aviadenovirus* contain linear, double-stranded, and non-segmented DNA, which is approximately 45 kb in length [23,24,25]. The coding region of the virus genome can be divided into an early region (E region) and a late region (L region) according to viral replication [26]. The late transcription gene 1 region (L1) of pigeon adenovirus encodes the nonstructural protein 52K. The 52K protein is composed of approximately 400 amino acids and is present in empty capsids, immature virions, and assembly intermediates. The 52K protein plays a critical role in the viral assembly process. This protein is responsible for the recognition of viral DNA and capsomers, thus ensuring the correct assembly and functional integrity of virions. Research has shown that in vivo, the 52K protein can directly bind to the packaging sequence through 331 amino acids at the N-terminus [27,28]. However, in vitro, it needs to first bind to the IVa2 protein through 173 amino acids at the N-terminal, and then they can interact with the sequence under IVa2 protein mediation to package the DNA into empty capsids.

High-resolution melting analysis (HRM) has been verified as a powerful diagnostic tool for detecting mutations, single nucleotide polymorphisms (SNPs), and epigenetic differences in clinical samples. HRM, as a real-time PCR-based platform, is based on the variance between the shapes of the melting curves and the difference between the melting temperatures (Tm) and offers many advantages for quantitative real-time PCR (qPCR) technology [29,30]. There are few systematic epidemiological studies on PiAdVs, and there are no reports on the use of the qPCR-HRM method for the simultaneous differentiation of PiAdV-1 and PiAdV-2 [31]. Here, we designed specific primers based on the nucleotide variations of the 52K gene of PiAdV-1 and PiAdV-2 and then established a qPCR-HRM platform for the detection and differentiation of PiAdV-1 and PiAdV-2. The specificity, sensitivity, and repeatability were evaluated and applied to the detection of clinical samples, aiming to provide specific, efficient, and sensitive techniques for the epidemiological investigation of PiAdV-1 and PiAdV-2.

## 2. Materials and Methods

### 2.1. Viruses and Controls

The viruses and controls used in this study, such as pigeon adenovirus 1 (PiAdV-1), pigeon adenovirus 2 (PiAdV-2), pigeon parvovirus (PiPV), pigeon paramyxovirus (PPMV), pigeon circovirus (PiCV), pigeon pox virus (PGPV), pigeon rotavirus (PiRVA), pigeon megrivirus (PiMV), and fowl adenovirus 4 (FAdV-4), which infects pigeons [32], were obtained from the Institute of Animal Husbandry and Veterinary Medicine, Fujian Academy of Agricultural Sciences.

Genomic DNAs (such as PiAdV-1, PiAdV-2, PiPV, PiCV, PGPV, and FAdV-4) or RNAs (including PPMV, PiRVA, PiMV) were extracted using a EasyPure^®^ Viral DNA/RNA Kit (TransGen Bioteck, Beijing, China), based on the manufacturer’s instructions. Then, the cDNAs (PPMV, PiRVA, PiMV) were obtained using EasyScript^®^ One-Step gDNA Removal and cDNA Synthesis SuperMix (TransGen Bioteck, Beijing, China).

### 2.2. Target Gene and Primer Design

According to the complete genome sequences of the PiAdV-1 and PiAdV-2 genes downloaded from GenBank, the 52K gene can be used as a primer design candidate region for the qPCR-HRM platform. Although the 52K gene shared only 71.1% nucleotide homology between PiAdV-1 and PiAdV-2, the target-amplified regions shared characteristic variations. The primers (P1P2-HRM1F and P1P2-HRM1R; Table 1) used for the qPCR-HRM platform were synthesized at Sangon (Sangon Biotech, Shanghai, China). The characteristic variations (175 bp) between PiAdV-1 and PiAdV-2 are listed in Table 2.

### 2.3. Preparation of Positive Standards

The PiAdV-1 and PiAdV-2 plasmids containing the target fragments of 52K were synthesized by Sangon Bioengineering Co., Ltd. (Shanghai, China). The recombinant plasmids of PiAdV-1 and PiAdV-2 (namely, T-PiAdV-1 and T-PiAdV-2) were evaluated via a NanoDrop2000. The T-PiAdV-1 and T-PiAdV-2 strains were subsequently diluted via easy dilution (TaKaRa, Dalian, China) 10 times, and the serial dilutions of T-PiAdV-1 (5.7 × 10^7^–5.7 × 10^0^ copies/μL) and T-PiAdV-2 (5.6 × 10^7^–5.6 × 10^0^ copies/μL) strains were subsequently used as templates for the qPCR-HRM platform.

### 2.4. Optimization of the qPCR-HRM Platform

To screen the optimal reaction system and reaction conditions for the qPCR-HRM assay, a 20 μL qPCR-HRM system was set up with different primer concentrations, annealing temperatures, and melting rates. The primer concentrations of the P1P2-HRM1F primer (10 μmol/L) and P1P2-HRM1R primer (10 μmol/L) (0.4, 0.5, 0.6, 0.7, 0.8, 0.9, and 1 μL, respectively) and the annealing temperatures (54–64 °C) of the qPCR-HRM assay were optimized. Using the optimized reaction system and conditions, three repetitions were set up for each reaction. The standard curve was established using the software of the qPCR machine. A dissolution curve was obtained to determine the coinfected samples via the qPCR-HRM platform using the software of the qPCR machine. All the samples were run in triplicate.

### 2.5. Sensitivity, Specificity, and Repeatability

For the sensitivity assay, four different concentrations of T-PiAdV-1 (5.7 × 10^0^–5.7 × 10^3^ copies/μL) and T-PiAdV-2 (5.6 × 10^0^–5.6 × 10^3^ copies/μL) were used and analyzed according to the optimized conditions to determine the limit of detection (LOD). For specificity of the qPCR-HRM platform, PiPV, PPMV, PiCV, PGPV, PiRVA, PiMV, and FAdV-4, which are present in pigeons, were used as controls. For repeatability, three different concentrations of T-PiAdV-1 (5.7 × 10^3^, 5.7 × 10^5^, and 5.7 × 10^7^ copies/μL) and T-PiAdV-2 (5.6 × 10^3^, 5.6 × 10^5^, and 5.6 × 10^7^ copies/μL) were selected. The coefficient of variation (CVs) of the qPCR-HRM platform between the intra-assay and inter-assay values were calculated using statistical analysis based on Ct values variations.

### 2.6. Clinical Sample Screening

To evaluate the prevalence of PiAdV-1 and PiAdV-2 in Fujian, Southeast China, a total of 70 feces (35 from racing pigeons and 35 from meat pigeons) were collected and tested. The samples were collected by the Avian Disease Diagnostic Laboratory of the Fujian Academy of Agricultural Sciences from October to December 2024. These clinical samples were placed in phosphate-buffered saline (PBS) and homogenized, and then the DNAs was extracted using a TIANamp Stool DNA Kit (Tiangen Biotech, Beijing, China). The extracted DNAs were tested via conventional PCR (cPCR) [33] and the established qPCR-HRM method.

## 3. Results

### 3.1. The Optimized qPCR-HRM Reaction

The results revealed that the optimal amplification reaction system was as follows: 10 μL of 2×SsoFast EvaGreen Supermix (Bio-Rad Biotechnology, Hercules, CA, USA), 1.0 μL of P1P2-HRM1F and P1P2-HRM1R, 1.0 μL of isolated DNA, and 7.0 μL of ddH_2_O. The optimal amplification program was as follows: 95 °C for 2 min; 40 cycles of 95 °C for 10 s and 60 °C for 20 s; and the melting curve parameters were set based on the protocol of the software of the qPCR machine.

### 3.2. Standard Curve of the qPCR-HRM Platform

**Figure 1:**
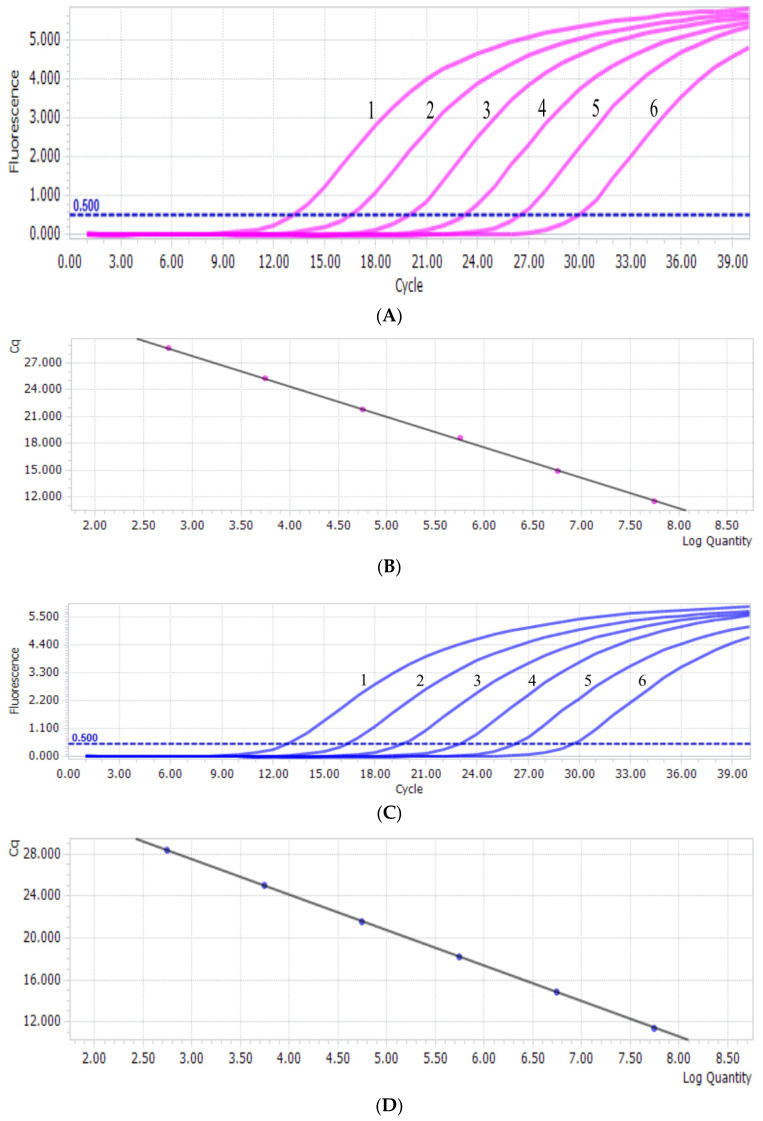
(**A**) PiAdV-1 amplification curve from the qPCR-HRM assay (1–6: the copy number concentration of T-PiAdV-1 was 5.7 × 10^7^–5.7 × 10^2^ copies/μL). (**B**) The standard curve of PiAdV-1 using the qPCR-HRM assay. (**C**) PiAdV-2 amplification curve from the qPCR-HRM assay (1–6: the copy number concentration of T-PiAdV-2 was 5.6 × 10^7^–5.6 × 10^2^ copies/μL). (**D**) The standard curve of PiAdV-2 using the qPCR-HRM assay.

The results revealed that T-PiAdV-1 had a linear relationship in the reaction from 5.7 × 10^2^ to 5.7 × 10^7^ copies/μL (Figure 1A). The standard curve regression equation was y = −3.4263x + 38.08, and the correlation coefficient R^2^ was 1.00 (Figure 1B). The linearity of T-PiAdV-2 was good using the dilution range from 5.6 × 10^2^ to 5.6 × 10^7^ copies/μL (Figure 1C). The standard curve regression equation was y = −3.4037x + 37.71, and the correlation coefficient R^2^ was 1.00 (Figure 1D).

### 3.3. HRM Analysis

**Figure 2:**
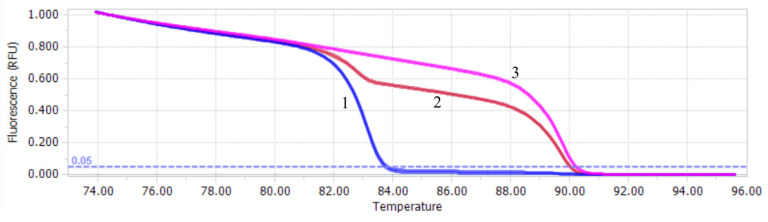
Melting profiles of the qPCR-HRM assay. 1: PiAdV-2; 2: mixed infection with PiAdV-1 and PiAdV-2; 3: PiAdV-1.

The results of the in vitro mixed sample test revealed that the qPCR-HRM method established in this study had a high discrimination ability for mixed samples and could clearly distinguish the infection of PiAdV-1 and PiAdV-2 according to the melting curve. The Tm of PiAdV-1 and PiAdV-2 were (89.44 ± 0.12) °C and (82.79 ± 0.09) °C. The coinfection rates of PiAdV-1 and PiAdV-2 in the qPCR-HRM platform are shown in Figure 2. The data showed that PiAdV-1, PiAdV-2, and coinfection with PiAdV-1 and PiAdV-2 produced significantly different dissolution curves.

### 3.4. Sensitivity, Specificity, and Repeatability

**Figure 3:**
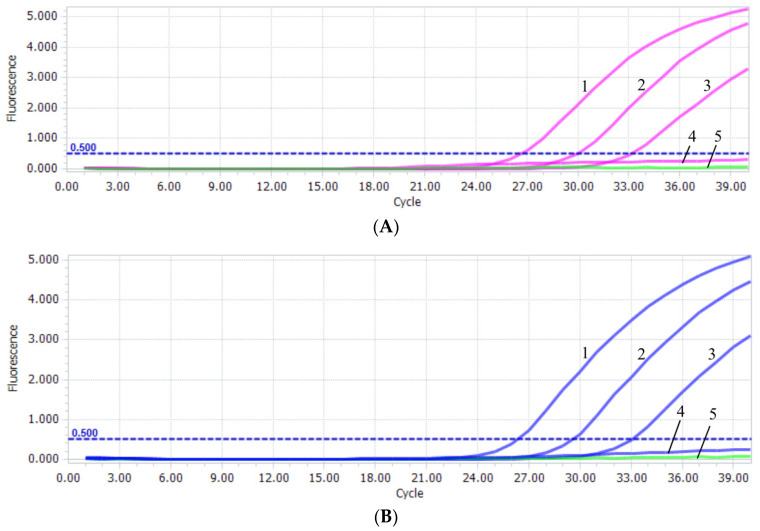
(**A**) PiAdV-1 sensitivity in the qPCR-HRM assay. 1–4: the copy number concentrations of T-PiAdV-1 was 5.7 × 10^3^–5.7 × 10^0^ copies/μL; 5: negative control. (**B**) PiAdV-2 sensitivity in the qPCR-HRM assay. 1–4: the copy number concentrations of T-PiAdV-2 were 5.6 × 10^3^–5.6 × 10^0^ copies/μL. 5: negative control.

**Figure 4:**
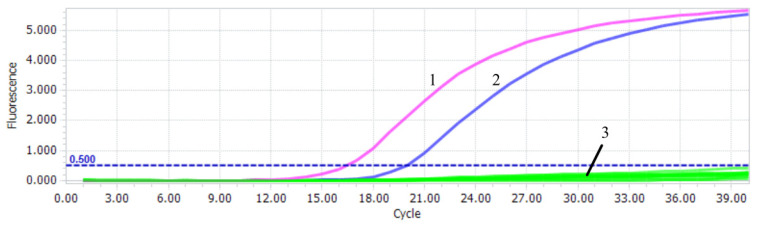
Specificity test of the qPCR-HRM assay. 1: PiAdV-1; 2: PiAdV-2; 3: PiPV, PPMV, PiCV, PGPV, PiRVA, PiMV, FAdV-4, and negative control.

The sensitivity test of PiAdV-1 and PiAdV-2 were 57 copies/µL (Figure 3A) and 56 copies/µL (Figure 3B), respectively. The specificity of the qPCR-HRM assay showed that the established assay could only detect positive amplification signals for PiAdV-1 and PiAdV-2, and did not show any positive amplification fluorescence signals for other pigeon-origin pathogens that occur in pigeons (such as PiPV, PPMV, PiCV, PGPV, PiRVA, PiMV, and FAdV-4) (Figure 4). The repeatability test data revealed that the intra-assay and inter-assay coefficients of variation were both less than 1.5% (Table 3), indicating that the qPCR-HRM assay had good repeatability and stability.

### 3.5. Detection of Clinical Samples

For the racing pigeon samples, two for PiAdV-1 (5.71%), five for PiAdV-2 (14.29%), and one for PiAdV-1 and PiAdV-2 coinfection (2.86%) were screened via the developed qPCR-HRM platform. Moreover, two for PiAdV-1 (5.71%), three for PiAdV-2 (8.57%), and one for PiAdV-1 and PiAdV-2 coinfection (2.86%) were screened via the cPCR method (Table 4). For the meat pigeon samples, only three for PiAdV-2 (8.57%), with no coinfection, were screened via the developed qPCR-HRM platform. Moreover, zero for PiAdV-1, two for PiAdV-2 (5.71%), and none for PiAdV-1 or PiAdV-2 coinfection were screened via the cPCR method (Table 4).

## 4. Discussion

As a contact infectious pathogen, pigeon adenovirus has a wide range of transmission routes and a high incidence rate, with the peak period of infection occurring from March to July each year. The virus can be transmitted vertically through pigeon breeding; it can also be transmitted horizontally, mainly in the form of fecal–oral transmission, in intensive breeding pigeon factories or pigeon racing shelters. Owing to poor breeding conditions, backwards management, and weak prevention awareness in some commercial meat pigeon and racing pigeon farms, the detection rate of pigeon adenovirus has steadily increased, causing serious economic losses to the pigeon industry [6,11,12,13,34]. At present, no efficient vaccine or specific treatment can be used for PiAdV disease prevention and control in endemic areas. Moreover, the etiology, transmission routes, and control measures for pigeon-related adenoviruses are still in the primary stage [35,36,37].

Laboratory diagnostic methods for adenovirus infection include clinicopathological features, histopathological examination, virus isolation, and the serological techniques including agar gel immunodiffusion (AGID), double immunodiffusion (DID), AGP tests, and enzyme linked immunosorbent assays (ELISA) [38,39], as well as restriction endonuclease analysis (REA), conventional PCR (cPCR), PCR-RFLP, loop-mediated isothermal amplification (LAMP), cross-priming amplification (CPA), recombinase polymerase amplification (RPA), qPCR, and HRM. A preliminary diagnosis is usually made on the basis of clinical symptoms, but further confirmation of PiAdV infection requires a combination of laboratory diagnostic techniques. ELISA had been widely used to detect the antibodies of pathogens and even can be used for type- or serotype-specific antibody detection [40,41,42]. Nowadays, a wide variety of DNA-based molecular methods, for the molecular epidemiology of pigeon diseases, has been reported based on their advantages of higher specificity and sensitivity. Quantitative real-time polymerase chain reaction (qPCR) is a widely used DNA-based method, which has the advantage of simultaneous amplification and quantification, and has been reported in recent years [43,44,45,46,47].

High-resolution melting (HRM) is a gene sequence analysis technique developed in recent years. High-resolution melting (HRM) combined with qPCR techniques (qPCR-HRM) can not only detect single nucleotide polymorphisms, indels, tandem repeats, methylation, and mitochondrial monomer types in genes but also be used for the mutation scanning and temperature calibration of fluorescence polymerase chain reaction apparatuses. With the popularization of fluorescence quantitative PCR instruments equipped with HRM analysis software, HRM technology has been widely used in laboratory pathogen detection and virus genotyping [29,30,48,49,50]. In addition, the EvaGreen dye used in this study is a novel green-fluorescent nucleic acid dye that has little ability to inhibit PCR amplification, is unlikely to cause nonspecific amplification, and has strong stability. Compared with other methods, the qPCR-HRM method is simple to perform, does not require gel electrophoresis after PCR, and truly achieves closed-tube operation, greatly avoiding the possibility of contamination. Therefore, this method is suitable for the large-scale rapid identification and diagnosis of infectious disease pathogens [51,52,53,54,55]. At present, there is no method for the simultaneous differential diagnosis of PiAdV-1 and PiAdV-2.

In this study, the qPCR-HRM platform established in this study could accurately detect single infection and coinfection of PiAdV-1 and PiAdV-2 with only a pair of primers and a single qPCR-HRM reaction. The melting peak Tm formed by PiAdV-1 was relatively high, while the melting peak Tm formed by PiAdV-2 was relatively low, and this difference in the melting peak Tm was approximately 6.65 °C. The method had strong specificity for PiAdV-1 and PiAdV-2 and had no cross-reaction with common pigeon-origin viruses (PiPV, PPMV, PiCV, PGPV, PiRVA, PiMV, and FAdV-4). The method had high sensitivity, and the minimum detection limits were 57 copies/µL and 56 copies/µL, respectively. The method has good repeatability, and the coefficients of variation from the intra-group repeatability test and inter-group repeatability test were both less than 1.5%.

An epidemiological survey from pigeon-origin fecal samples demonstrated that PiAdV-1 and PiAdV-2 coinfection (with a positive rate of 2.86%) was screened in racing pigeons but no coinfection was found in meat pigeons, suggesting that further epidemiological investigation of PiAdV-1 and PiAdV-2 in pigeons should be performed, which will be beneficial for the ecology and pathogenic mechanism of pigeon adenoviruses. Additionally, we also found all cPCR positive samples were also followed with positive signals using the established qPCR-HRM platform with these data indicating that qPCR or qPCR-HRM detection was more sensitive than cPCR.

## 5. Conclusions

In conclusion, we first developed a powerful tool for the simultaneous detection and differentiation of PiAdV-1 and PiAdV-2 using the qPCR-HRM platform, which offers the advantage of qPCR technology. We also present the first evidence of PiAdV-1 and PiAdV-2 coinfection in pigeons.

## Figures and Tables

**Table 1:** Primers used for the qPCR-HRM platform.

Viruses	P1P2-HRM1F (5′-3′)	Position	P1P2-HRM1R (5′-3′)	Position	Length (bp)
PiAdV-1	AAGACGGYTACCCGAG	880–896 ^A^	ACATGTAGTCCGCGTCAGTCA	1034–1054 ^A^	175
PiAdV-2		874–890 ^B^		1028–1048 ^B^	

A, the length of the 52K sequence of PiAdV-1 is 1182 bp, which corresponds to the position of the 52K sequence of PiAdV-1. B, the length of the 52K sequence of PiAdV-2 is 1164 bp, which corresponds to the position of the 52K sequence of PiAdV-2.

**Table 2:** Variations in the target region of 52K between PiAdV-1 and PiAdV-2.

**Position**	**1–60**
PiAdV-1	AAGAACGGCTACCCGAG CATGGCGCAGATGGCGAAAGCGCAGGAGTTCTTTTTTCGCGTG
PiAdV-2	AAGAACGGTTACCCGAG TATGGGTCAGATGGCTAAAGCTCAAGAGTTTTTCTTCAGAATC
**Position**	**61–120**
PiAdV-1	ATGGAGGCCATCCTGGACCTGGGCGTGCAGTTGGGCATTTACCACAACCACCCGGTGCCG
PiAdV-2	ATGCAAGCAATACTGGATTTAGGAGTGCAGTTAGGGGTTTACAACAATCGTCCGGTACCG
**Position**	**120–175**
PiAdV-1	TACCGGCAGAAGCGGGCGAGCGAGCTGCCGCAGCTGACTGACGCGGACTACATGT
PiAdV-2	TTCCGTCAAAAGAGGGCGTCTGATATTCCGCAGATGACTGACGCGGACTACATGT

The variation in the target region (175 bp) of 52K between PiAdV-1 and PiAdV-2 is listed with the primer sequences underlined, and the variations in 52K between PiAdV-1 and PiAdV-2 are marked in red.

**Table 3:** Repeatability data of the qPCR-HRM assay.

Virus	Concentration of Plasmid Standards (Copies/μL^−1^)	Intra-Assay Reproducibility		Inter-Assay Reproducibility	
		Cq ± SD	CV/%	Cq ± SD	CV/%
PiAdV-1	5.7 × 10^7^	11.56 ± 0.12	1.06	11.63 ± 0.17	1.42
	5.7 × 10^5^	18.44 ± 0.09	0.47	18.59 ± 0.13	0.72
	5.7 × 10^3^	25.29 ± 0.13	0.49	25.39 ± 0.20	0.79
PiAdV-2	5.6 × 10^7^	11.35 ± 0.06	0.57	11.53 ± 0.16	1.40
	5.6 × 10^5^	18.24 ± 0.06	0.31	18.39 ± 0.12	0.66
	5.6 × 10^3^	25.01 ± 0.11	0.44	25.11 ± 0.27	1.09

**Table 4:** Detection and differentiation of PiAdV-1 and PiAdV-2 in clinical samples.

Species	Number	HRM						cPCR					
		PiAdV-1		PiAdV-2		Coinfection		PiAdV-1		PiAdV-2		Coinfection	
		P	R	P	R	P	R	P	R	P	R	P	R
Racing pigeon	35	2	5.71	5	14.29	1	2.86	2	5.71	3	8.57	1	2.86
Meat pigeon	35	0	0	3	8.57	0	0	0	0	2	5.71	0	0

Abbreviations: P means the number of positive samples, and R means the positive ratio (/%).

## Data Availability

The original contributions presented in this study are included in the article. Further inquiries can be directed to the corresponding authors.
